# Ongoing Sign Processing Facilitates Written Word Recognition in Deaf Native Signing Children

**DOI:** 10.3389/fpsyg.2022.917700

**Published:** 2022-08-05

**Authors:** Barbara Hänel-Faulhaber, Margriet Anna Groen, Brigitte Röder, Claudia K. Friedrich

**Affiliations:** ^1^Department of Special Education, Universität Hamburg, Hamburg, Germany; ^2^Department of Psychology, Lancaster University, Lancaster, United Kingdom; ^3^Biological Psychology and Neuropsychology, Universität Hamburg, Hamburg, Germany; ^4^Department of Developmental Psychology, University of Tübingen, Tübingen, Germany

**Keywords:** sign language, ERPs, lexical processing, deaf children, reading, German Sign Language (DGS)

## Abstract

Signed and written languages are intimately related in proficient signing readers. Here, we tested whether deaf native signing beginning readers are able to make rapid use of ongoing sign language to facilitate recognition of written words. Deaf native signing children (mean 10 years, 7 months) received prime target pairs with sign word onsets as primes and written words as targets. In a control group of hearing children (matched in their reading abilities to the deaf children, mean 8 years, 8 months), spoken word onsets were instead used as primes. Targets (written German words) either were completions of the German signs or of the spoken word onsets. Task of the participants was to decide whether the target word was a possible German word. Sign onsets facilitated processing of written targets in deaf children similarly to spoken word onsets facilitating processing of written targets in hearing children. In both groups, priming elicited similar effects in the simultaneously recorded event related potentials (ERPs), starting as early as 200 ms after the onset of the written target. These results suggest that beginning readers can use ongoing lexical processing in their native language – be it signed or spoken – to facilitate written word recognition. We conclude that intimate interactions between sign and written language might in turn facilitate reading acquisition in deaf beginning readers.

## Introduction

There is an ongoing debate on how deaf individuals commanding a signed language acquire literacy (Perfetti and Sandak, 2000; Holmer et al., 2016). Written languages typically are based on spoken languages and signed languages do not share relevant phonology or orthography with written languages. Therefore, deaf individuals can typically not use direct form links between sign and written language. Here, we tested whether emerging literacy in deaf children is closely connected to sign language processing at the word level nevertheless. From a neurocognitive perspective, we investigated whether young signers can exploit aspects of rapid sign processing to foster written word recognition and whether they do so similar to young hearing readers exploiting aspects of rapid spoken word processing.

Similar to hearing individuals processing sequentially unfolding speech in an incremental fashion, signing individuals process sequentially unfolding signs gradually. As soon as hearing individuals have heard some speech sounds, they have available memory representations of words that temporally match the input, and they sequentially exclude those words that no longer match the unfolding input thereafter (e.g., Allopenna et al., 1998; Dahan et al., 2001). Although fundamentally different from phonology in spoken languages, signed languages have a sequential, decomposable phonological structure as well. Typically, handshape, location, movement, palm orientation, and non-manual cues like facial gestures (including mouth movements) are considered as phonological sign language units which define individual signs and unfold over time (Sandler and Lillo-Martin, 2006; Papaspyrou et al., 2008; Brentari, 2011). Comparable to listeners recognizing spoken words, signers use the sequential nature of signs to activate corresponding memory representations even before a signer has completed a sign (Grosjean, 1981; Clark and Grosjean, 1982; Emmorey and Corina, 1990).

In hearing readers, incremental processing of spoken words can immediately modulate the processing of written words. Respective processing links between both domains are exemplified by priming studies, which typically combine spoken primes (complete words or word onsets) and written targets (for an overview see Zwitserlood, 1996). A direct repetition of spoken and written words [“pepper” – *pepper* (here and in the following, italics represent written stimulus materials)] immediately facilitates processing of written target words (compared to unrelated prime-target pairs like “pepper” – *window*) in hearing adults (Holcomb et al., 2005) and in hearing children (Reitsma, 1984; Sauval et al., 2017). Facilitated processing has been observed already when only spoken word onsets were presented as primes (e.g., “can” – candy or “ano” – anorak; Soto-Faraco et al., 2001; Spinelli et al., 2001; Friedrich et al., 2004a,b, 2008, 2013; Friedrich, 2005).

Event-related potentials (ERPs) recorded in priming studies indicate that the processing initiated by spoken word onsets taps early aspects of the processing of immediately following written target words in hearing adults. Two ERP deflections are typically obtained when spoken word onsets are used to prime written target words: Prime-target overlap in phonology consistently elicited left-lateralized more positive-going ERP amplitudes (the so-called P350 effect in word onset priming), and reduced N400 amplitudes with central distribution (compared to unrelated targets, respectively; Friedrich et al., 2004a,b, 2013; Friedrich, 2005). Both effects start 200–300 ms after the onset of a written target word, a time window that is associated with access to stored word representations (e.g., Grainger et al., 2006; Holcomb and Grainger, 2006, 2007). Based on the intimate phonological form relationship between spoken and written words in alphabetic writing systems, links between spoken and written language processing might already originate at the level of phonological representations (e.g., Ferrand and Grainger, 1992; Grainger et al., 2006; Pattamadilok et al., 2017), but might also relate to the level of word form representations. The question emerges whether links between written language and sign language, which do not connect via grapheme-phoneme correspondence at the surface level, originate at the level of word form representations as well.

Previous priming research showed that signing adults implicitly activate signs and their respective phonological forms when they are reading written words [e.g., ASL while reading English words: Morford et al., 2011, 2014; Meade et al., 2017; Quandt and Kubicek, 2018; DGS while reading German words: Kubus et al., 2015; Hong Kong Sign Language (HKSL) while reading Cantonese words: Thierfelder et al., 2020]. These studies exploited pairs of written words that were not related in the written or phonological domain in a given spoken language, but shared sign units in a respective sign language, such as MOVIE and PAPER sharing location and handshape in ASL (here and in the following, capitals denote signed stimulus materials), but no speech sounds in spoken English. When deaf signing participants had to detect semantic similarities for pairs of written words, implicit phonological priming of the underlying signs speeds responding and, vice versa, decisions about semantic differences for pairs of written words slowed down when they overlapped in sign phonology (for ASL: Morford et al., 2011, 2014; for DGS: Kubus et al., 2015). In addition, Deaf native signers show phonological similarity effects in ASL when they have to recall lists of English written words (Miller, 2007).

By using online neurocognitive measures, previous ERP studies with signing adults suggested intimate links between sign word processing and the processing of written words. This was attested by unimodal priming studies combining either signed prime-target pairs (Lee et al., 2019; Hosemann et al., 2020) or written prime-target pairs (Meade et al., 2017). In the signed priming studies, phonological relation in the written domain modulated responses to phonologically unrelated sign pairs like BAR – STAR (with no phonological relation in ASL, but phonological relation in written English; e.g., Lee et al., 2019). Here, N400 effects starting 325 ms after target word onset were obtained (however, interpretation of the onset of ERP effects for signed targets is hampered by variation regarding the sequential nature and respective temporal characteristics of the continuously unfolding signs). In priming studies with written stimuli, phonologically and orthographically unrelated written word pairs like *gorilla – bath* were related in their sign language translations like GORILLA – BATH (sharing handshape and location in the corresponding ASL signs; Meade et al., 2017). Written prime-target pairs overlapping in sign phonology elicited a N400 effect starting 300 ms after target word onset. In the present study, we tested whether signing children are linking sign word processing to written word processing as early as adult signers do.

So far, very little is known about aspects of sign language processing and their links to reading in children who have acquired a sign language. Two phonological priming studies have suggested that deaf children, who were native signers of the Sign Language of the Netherlands (NGT), applied incremental phonological processing to signs (Ormel et al., 2009) and that signs are tightly associated with written words (Ormel et al., 2012). In their first study, Ormel and colleagues tested 8–12-year-old deaf children with picture-sign pairs. Children were asked whether the picture and the sign matched (picture verification task). Some unrelated sign-picture pairs, such as DOG and CHAIR, shared sign phonology in NGT (location and movement), while other sign-picture pairs were unrelated in that respect. As in previous work with deaf adults, in deaf children implicit phonological priming of the signs inhibited responding in cases where the sign-picture pairs were unrelated. In a follow-up study, Ormel and colleagues investigated 9–11-year-old deaf children in a picture-word verification task. In that study, NGT translation of the Dutch word and the sign for the picture were either phonologically related or not. Again, children indicated mismatches more slowly when word-picture pairs implicitly overlapped in sign phonology (compared to unrelated pairs). Recently, co-activation of ASL and written English in deaf signing children (mean age of 12.9 years) has been investigated by using a semantic judgment task for written words (Villwock et al., 2021). The children were faster to make “yes” decisions (the words are semantically related) when the ASL translations were phonologically related. As in previous studies for deaf adults (Morford et al., 2011), a subset of the presented semantically related and unrelated word pairs shared sign phonology in ASL. Children were faster to respond to written word pairs with phonological relations in ASL. Consistent with the results of Ormel et al. (2012) this indicates that children have sign language phonology available while they are reading.

**FIGURE 1:**
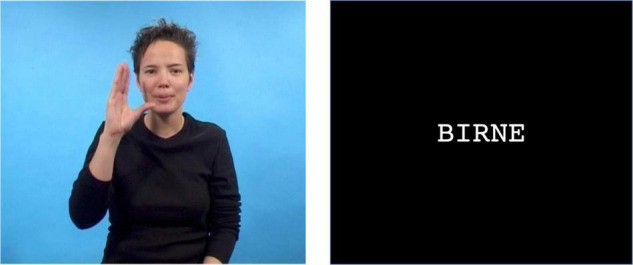
Illustration of one trial in the overlapping sign fragment – written word condition: The sign fragment “BI” for BIRNE (pear) is followed by the overlapping written word *Birne* (pear).

In the present study, we use online neurocognitive measures to investigate the temporal processing dynamics underlying interactions between sign and written language processing in deaf signing beginning readers. By recording ERPs to targets in word onset priming, we aimed to uncover whether beginning readers use incremental processing of sign onsets (deaf native signing children) similarly to incremental processing of spoken word onsets (hearing children) to foster ongoing written word processing. Deaf and hearing children were matched on reading skills. Deaf beginning readers saw videos of sign word onsets (primes), which were followed by written words (targets). Hearing beginning readers watched and heard a speaker articulating word onsets (primes), which were followed by written words (targets). Both groups were asked to decide whether the written target was a possible German word. The crucial comparison within a group was between responses to targets in the condition where prime and target were related [Overlapping condition; e.g., KU^1^ – *Kuchen* (Engl. cake) and “ku” – *Kuchen*, respectively] versus the Unrelated condition (e.g., WE – *Kuchen* and “we” – *Kuchen*, respectively). An example trial (Overlapping condition) with a sign prime with a sign prime, followed by a written word target is provided in Figure 1.

Based on earlier studies using spoken-written word onset priming with hearing adults (Friedrich et al., 2004a,b, 2008, 2013; Friedrich, 2005), we expected to find P350 and N400 effects preceding behavioral responses (lexical decisions). If beginning readers exploit ongoing processing in their native language as rapidly as experienced adult readers, ERP effects should start 200 ms after target word onset (for hearing adults: e.g., Friedrich et al., 2004a,b, 2008, 2013; Friedrich, 2005; Grainger et al., 2006; for deaf signing adult readers: e.g., Gutierrez-Sigut et al., 2017).

## Materials and Methods

### Participants

Data from fourteen congenitally deaf children (hearing threshold >90 dB in the better ear; seven girls) who had learned sign language from birth from their deaf parents (“native signers”) and fourteen typically developing, hearing, children (four girls) with hearing parents (“controls”) were included in the study. We recruited deaf children from Schools for Deaf and Hard of Hearing in Germany which ran a bilingual (German and DGS) curriculum at the time. A control group of hearing children was then recruited by matching levels of word reading comprehension across groups. The hearing children were monolingual speakers of German from local primary schools in the city of Hamburg. An additional three native signers and four controls originally participated in the study, but their data could not be included in analyses because of low quality of EEG data as a result of excessive movement by the participant (two native signers; two controls), refusal by the participant to complete the reading test (one native signer), low performance on the reading test (one control) or technical failure during EEG recording (one control). None of the children had any neurological disease or learning difficulties. We obtained written informed parental consent for all children.

Data from native signers was collected first. Based on their performance on a normed German word reading test for beginning readers (ELFE 1–6, word reading comprehension subtest, Lenhard and Schneider, 2006), a younger control group was then recruited to ensure similar levels of word reading comprehension across groups. In this subtest, a picture was presented together with four written words. The child was asked to underline the word that matched the picture. Reported are raw scores, which consist of the total number of correct responses within a time window of 3 min [native signers: *M* = 35.9, *SD* = 9.6; controls: *M* = 35.9, *SD* = 7.1; *t*(26) = 0, *p* = 1, Cohen’s *d* = 0]. Note, that the timeline of reading development for deaf and hearing readers differs due to the patterns of language exposure and the access to language input in the two populations (see e.g., Mayberry et al., 2011; Miller and Clark, 2011; Trezek et al., 2011). As a result, the control group was significantly younger than the group of native signers [native signers: *M* = 10.7 years; *SD* = 18 months; controls: *M* = 8.8, *SD* = 10 months; *t*(26) = 4.16, *p* < 0.001, Cohen’s *d* = 1.57]. The two groups did not differ in non-verbal cognitive abilities [native signers: *M* = 68.1, *SD* = 25.9; controls: *M* = 62.8, *SD* = 27.1; *t*(26) = −0.53, *p* = 0.603, Cohen’s *d* = 0.20]. Raven’s Colored Progressive Matrices (Raven, 1936) were used as a measure of non-verbal cognitive ability. Percentile scores in relation to norms for German children are reported (Bulheller and Häcker, 2002).

### Stimuli

We used 80 concrete common nouns (see Supplementary Material), selected to be known to young children. As no lexical developmental scale for the acquisition of DGS exists, we checked the nouns with the CDI-ASL (Anderson and Reilly, 2002) and CDI-BSL (Woolfe et al., 2010). While targets consisted of complete written words, primes consisted of onsets of signs/spoken words. Following work by Friedrich et al. (2009) and Schild et al. (2011), spoken word onsets consisted of the first syllable of a respective target word. We created the spoken word onsets (fragments) by filming a hearing male actor speaking the complete words in front of a blue screen. Spoken fragments were created by editing each word after the first syllable. The sign stimuli were created by filming a female deaf native signer of DGS while she produced each noun in DGS in front of a blue screen. The signer was a professional employee of a sign language movie company.

As no grading system exists which would have allowed us to determine the point of uniqueness for DGS signs, we created the sign fragments by taking into account theories on sign phonology. Hereby, the parameter *location* is proposed to be equivalent to a syllable onset and *movement* and *location* properties serve as the skeletal structure for syllable-like units (for a more differentiated analysis of syllables in signs see Sandler, 1989; Brentari, 1998). The combination of movement and location has been shown to result in phonological effects on lexical retrieval which are similar across language modalities (Gutierrez et al., 2012a,b). Taking this as the basis for the production of the sign fragments, a deaf native signer cut each complete sign video at that point in time, when the hands were in the correct position in terms of *location*. Since sign phonological segments are expressed simultaneously, all sign fragments presented the correct *handshape* and a very brief *movement* (*M* = 52.94 ms). Sign fragments (*M* = 1418 ms, *SD* = 108) were on average longer in duration than spoken fragments (*M* = 1050 ms, *SD* = 19).

In order to determine if these fragments were ambiguous sign onsets, we presented them to two deaf native and two deaf near-native signing adults whom we asked to complete each sign fragment as rapidly as possible. Out of 80 sign fragments, 43 resulted in the production of the intended complete sign by all participants (“unambiguous sign word onsets”). In contrast, the remaining 37 sign fragments received at least one different completion than the target sign it was created from (“ambiguous sign word onsets,” marked by an asterisk in the Supplementary Material). Because we needed all trials in the ERP experiment and were limited in the choice of signs due to other criteria (e.g., that they were concrete nouns, known by young children) we decided to include all trials in the ERP analyses. However, for the reaction times, we additionally analyzed the responses for ambiguous vs. unambiguous word onsets separately (see section “Results”).

For each participant, half the concrete nouns (i.e., 40) were used as targets in the Overlapping condition, and the other half were used as targets in the Unrelated condition. Allocation of targets to condition was counterbalanced across participants in both groups. The same primes were used to precede targets in both of these conditions. For example, a participant was presented with the prime followed by the target in the Overlapping condition (e.g., KU/“ku” – *Kuchen* [Engl. cake]) in one block and that same prime followed by the target for the Unrelated condition (e.g., KU/“ku” – *Wecker* [Engl. alarm clock]) in a different block. Additionally, 20 trials with pseudowords were presented for the lexical decision task. Pseudowords were created that differ only in the last one or two letters from the words. In 10 of those trials the prime and pseudoword showed overlap (e.g., AU/“au,” *Aune* [pseudoword derived from Auto, Engl. car]); in the remaining 10 trials, the prime and the pseudoword were unrelated (e.g., prime for HUNG/”hung,” *Namel* [pseudoword derived from Name, Engl. name]).

### Procedure

All participants were tested individually, in a quiet room in their school (native signers) or at the university (controls). After completing the reading and non-verbal cognitive ability tests, the EEG recording cap was fitted and the child was seated behind a computer.

Presentation^®^ software (Neurobehavioral Systems, Inc., Berkeley, CA, United States)^2^ was used to control stimulus presentation and record behavioral responses. All visual stimuli were presented on a computer screen placed approximately 40 cm in front of the participant, with videos of sign and spoken stimuli being presented at natural speed, and at a size of 21.4 cm by 17.1 cm, on a black background showing the face and torso of the speaker. Written stimuli were in white capital letters (font: Courier, font size: 41) on a black background. Auditory stimuli were presented to controls through speakers positioned directly to the right and the left of the computer screen.

Each trial began with the presentation of the fixation picture for 1,000 ms at the center of the screen, which participants were asked to fixate on whenever it appeared. The prime was presented, followed by a blank screen for 450 ms, before the target was presented. Subsequently, participants were asked to press the space bar only if they believed the target was a possible written word in German. A response was followed by a feedback stimulus (2,000 ms in duration) consisting of a smiley for correct and a picture of a ghost for incorrect responses. The next trial started after a 1,500 ms inter-trial interval (from response onset) during which the screen was blank. If participants did not respond within 5,000 ms, the task continued with the inter-trial interval regardless.

Trials were presented in one of two pseudo-random orders, and in blocks of 10 with short breaks in between. A set of 10 practice trials preceded the experimental blocks. Trial order and response hand were counterbalanced across participants in both groups. The total duration of the experiment was about 60 min (including breaks).

### Event Related Potential Recordings and Analysis

**FIGURE 2:**
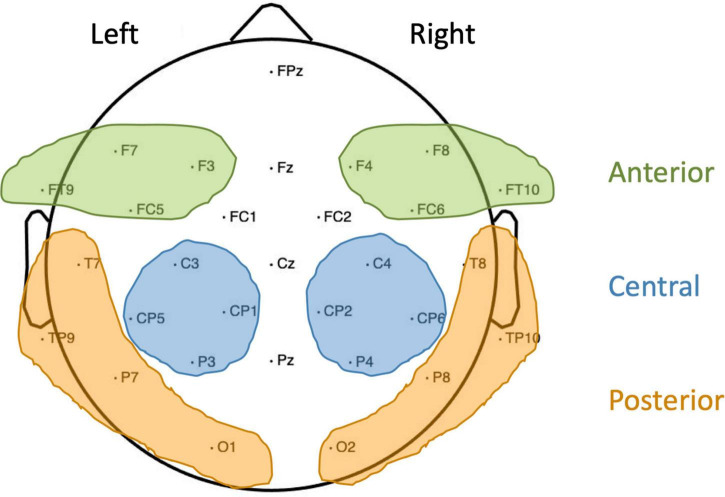
Overview of electrodes used and regions of interest formed. For the P350, left and right anterior (shaded green) and posterior (shaded yellow) regions were used. For the N400, left and right central regions (shaded blue) were used.

The continuous electroencephalogram (EEG; 500 Hz/22 bit sampling rate, 0.01–100 Hz bandpass) was recorded from 30 Ag/AgCl active electrodes (Brain Products) mounted into an elastic cap (Easycap) according to the 10–20 system. Additionally, electrodes F9 and F10 (positioned close to the outer canthi of the left and right eye) were used to monitor horizontal eye movements, while two further electrodes were attached below the eyes to record vertical eye movements, all referenced to the nose. A left frontal scalp electrode (AF3) served as ground. Off-line analysis was performed using BESA-Research software (MEGIS Software GmbH; Version 5.3): the EEG was re-referenced to an average reference, eye artifacts were corrected using surrogate Multiple Source Eye Correction by Berg and Scherg (1994), and noisy trials were manually excluded. If an electrode was noisy throughout a substantial part of the recording, this electrode was interpolated. In controls, for two children no electrodes were interpolated, for three children one electrode was interpolated, for seven children two electrodes were interpolated and for another two children, three electrodes were interpolated. In native signers, for eight children no electrodes were interpolated, for three children one electrode was interpolated and in another three children two electrodes were interpolated. A minimum of 22 artifact-free trials was included in each condition per child. Controls (*M* = 31.11, *SD* = 5.05) did not differ from native signers (*M* = 32.21, *SD* = 3.30) in the average number of artifact-free trials included per condition, *t*(26) = 0.336, *p* = 0.74, *d* = 0.26. Event-related potentials (ERPs) were computed for the target words with correct responses, starting from the beginning of the presentation of the written word up to 1,000 ms post-stimulus onset. The ERPs were baseline corrected to a 200 ms pre-stimulus period. The dependent variable for the ERPs was the mean amplitude for each participant in the Overlapping and the Unrelated condition across regions of interest and time windows informed by previous work (Friedrich et al., 2004a,b, 2013; Friedrich, 2005; Schild et al., 2011). For the P350, regions of interest were: left anterior (F7, F3, FT9, and FC5), right anterior (F4, F8, FC6, and F10), left posterior (T7, TP9, P7, and O1) and right posterior (T8, TP10, P8, and O2). For the N400, regions of interest were left central (C3, CP5, CP1, and P3) and right central (C4, CP2, CP6, and P4). Regions of interest are illustrated in Figure 2. Time windows for both the P350 and the N400 were 200–400 and 400–600 ms post-stimulus onset.

## Results

### Behavioral Responses

**FIGURE 3:**
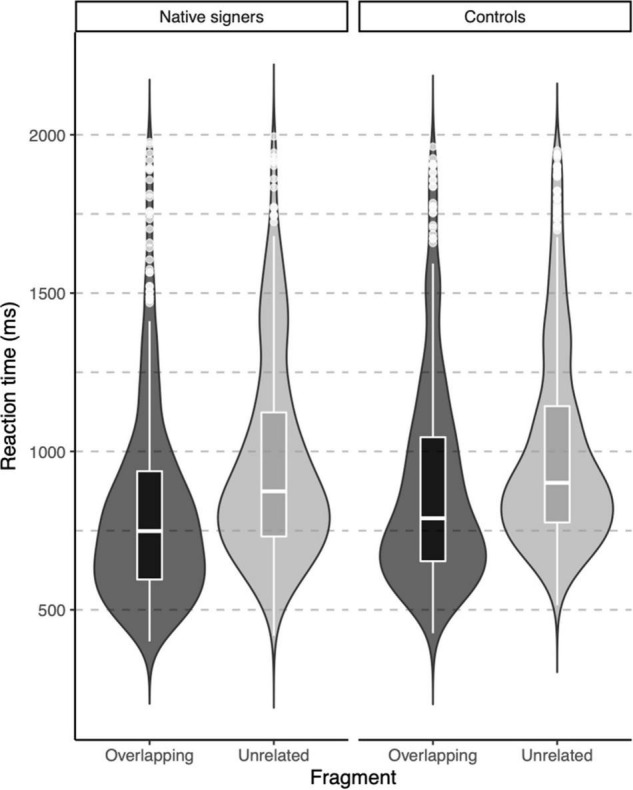
Violin-boxplots depicting reaction times (in ms) for correct responses for Native signers (left) and Controls (right) for Overlapping (dark gray) and Unrelated (light gray) fragments.

**FIGURE 4:**
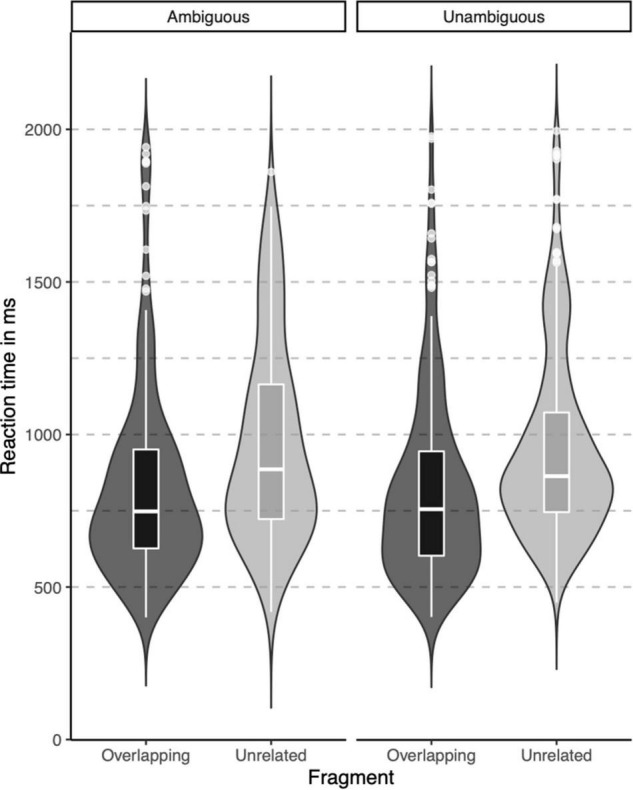
Violin-boxplots depicting reaction times (in ms) for correct responses in Native signers only, for Ambiguous (left) and Unambiguous (right) fragments.

In the lexical decision task, both groups were highly accurate in identifying words (percentage button presses in response to word targets), but native signers (*Mdn* = 97.5%) were slightly less accurate than controls (*Mdn* = 100%), *U* = 46, *p* = 0.012. The native signers additionally more often identified pseudowords as words (percentage button presses in response to pseudowords; Native signers: *Mdn* = 35%, Controls: *Mdn* = 17.5%, *U* = 116, *p* = 0.042). Reaction times were only analyzed for correct responses to word targets. In Figure 3, violin-boxplots depict reaction times across groups and word onsets. As the reaction times showed considerable positive skew, values were log-transformed (using the natural logarithm) before we conducted an ANOVA with Group (Native signers vs. Controls) as a between-subject factor and Word Onset (Overlapping vs. Unrelated) as a within-subject factor. The effect of Group was not significant [*F*(1,26) = 1.42, *p* = 0.244], neither was the interaction with Group [*F*(1,26) = 1.49, *p* = 0.233]. Crucially, the main effect of Word Onset was significant [*F*(1,26) = 107.27, *p* < 0.001]. Both native signers (Overlapping: *M* = 817 ms, *SD* = 311 ms; Unrelated: *M* = 962, *SD* = 314) and controls (Overlapping: *M* = 888 ms, *SD* = 325 ms; Unrelated: *M* = 1002, *SD* = 314) were faster to respond when prime and target overlapped than when they were unrelated. In native signers, an additional ANOVA with Word Onset (Overlapping vs. Unrelated) and Predictability (Ambiguous vs. Unambiguous) as within-subject factors resulted in a significant main effect of Word Onset [*F*(1,13) = 43.53, *p* < 0.001]. Neither the main effect of Predictability *F*(1,13) = 0.16, *p* = 0.700, nor the interaction with Predictability *F*(1,13) = 0.00, *p* = 0.950 was significant. Native signers were faster to respond when prime and target overlapped than when they were unrelated, both for ambiguous word onsets (Overlapping: *M* = 829 ms, *SD* = 314 ms; Unrelated: *M* = 971, *SD* = 326) and unambiguous word onsets (Overlapping: *M* = 823 ms, *SD* = 308 ms; Unrelated: *M* = 956, *SD* = 307; see Figure 4).

### Event-Related Potentials

**FIGURE 5:**
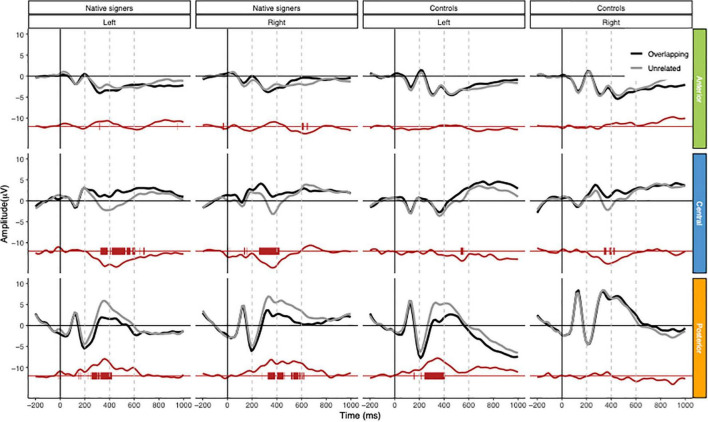
Grand average waveforms for Native signers (left two columns) and Controls (right two columns) for the Overlapping (black line) and Unrelated (gray line) fragments across regions of interest (Anterior, Central, Posterior × Left, Right). In red, the difference wave (Unrelated – Overlapping) as well as an indication of a significant difference from zero (point-by-point; *p* < 0.01) for the difference waves. Vertical dashed gray lines indicate measurement windows.

**FIGURE 6:**
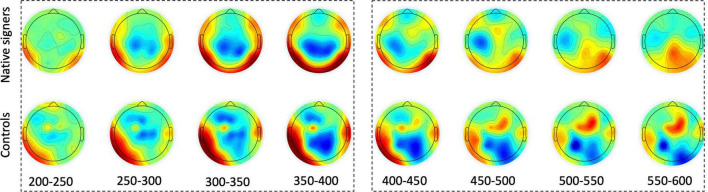
Topographical voltage maps of the difference waves (Unrelated – Overlapping) for Native signers (upper panels) and Controls (lower panels) between 200 and 600 ms post-stimulus in 50 ms increments. Dashed boxes indicate time windows used for measurement. Green indicates zero, colors toward dark red indicate a positive difference, colors toward dark blue indicate a negative difference. Nose is at the top.

**FIGURE 7:**
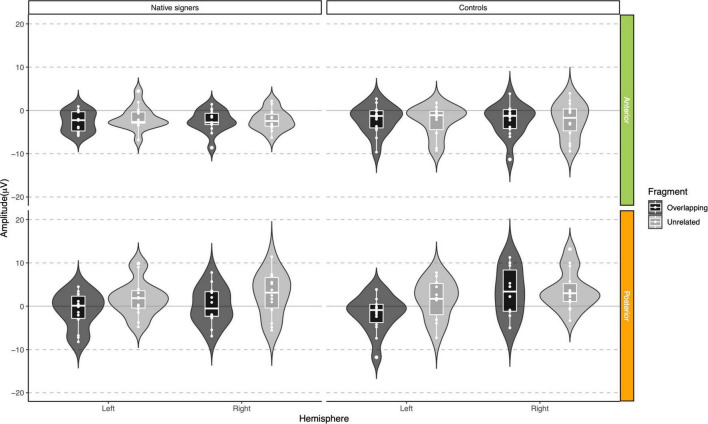
Violin-boxplots depicting mean amplitude for the P350/positivity in the 200–400 ms time window for Native signers (left) and Controls (right) for the Unrelated (light gray) and Overlapping (dark gray) fragments across regions of interest.

**FIGURE 8:**
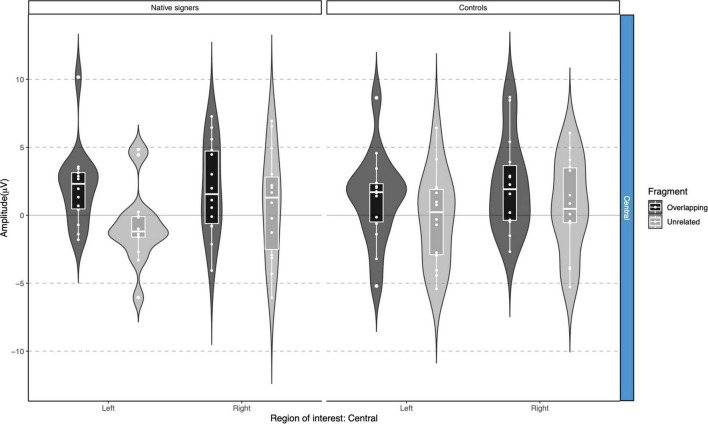
Violin-boxplots depicting mean amplitude for the P350/positivity in the 400–600 ms time window for Native signers (left) and Controls (right) for the Unrelated (light gray) and Overlapping (dark gray) fragments across regions of interest.

**FIGURE 9:**
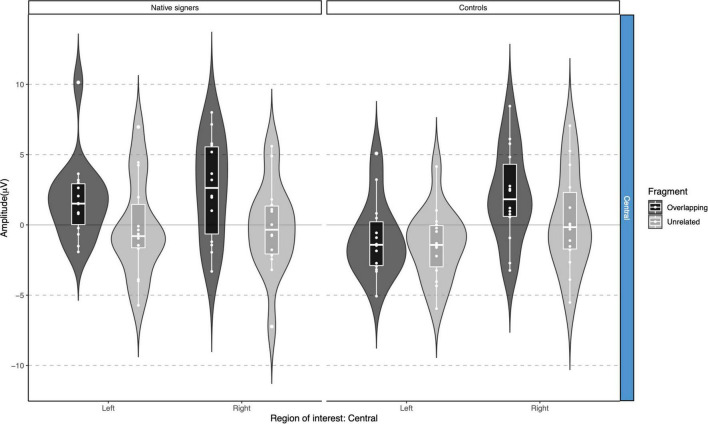
Violin-boxplots depicting mean amplitude for the N400/negativity in the 200–400 ms time window for Native signers (left) and Controls (right) for the Unrelated (light gray) and Overlapping (dark gray) fragments across regions of interest.

**FIGURE 10:**
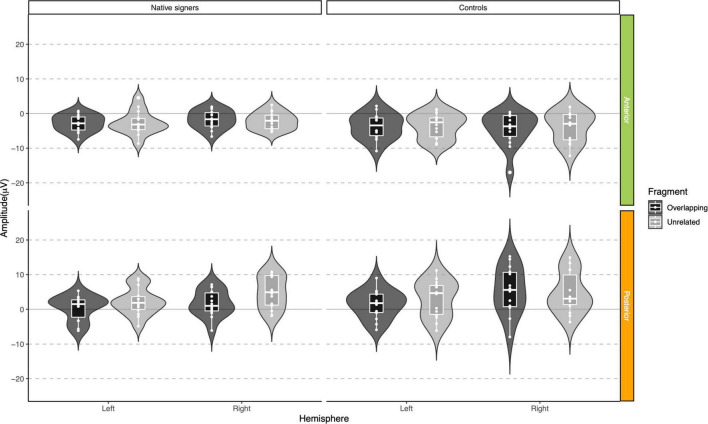
Violin-boxplots depicting mean amplitude for the N400/negativity in the 400–600 ms time window for Native signers (left) and Controls (right) for the Unrelated (light gray) and Overlapping (dark gray) fragments across regions of interest.

Grand average waveforms across word onsets (Overlapping vs. Unrelated) as well as difference waves (Unrelated – Overlapping) for each of the regions of interest (Anterior, Central and Posterior) and hemispheres (Left vs. Right) in both groups (Controls vs. Native signers) are presented in Figure 5. Figure 6 shows topographical voltage maps of the difference waves across the 200–400 ms and 400–600 ms time windows for both groups. A posterior positivity, which we relate to the P350 effect, was visible in both groups. This effect was left-lateralized in controls, whereas it was bilaterally distributed in native signers. At central regions, a bilateral negativity, which we relate to the N400, was evident in both groups. Central tendencies and distributions of mean amplitudes across conditions and groups for the left and right hemispheric regions of interest are presented in Figures 7, 8, for the P350/positivity and Figures 9, 10 for the N400/negativity.

For the P350 effect, a repeated-measures ANOVA with Word Onset (Overlapping vs. Unrelated), Region (Anterior vs. Posterior) and Hemisphere (Left vs. Right) as within-subject factors and Group (Controls vs. Native signers) as a between-subject factor was separately conducted for the lateral regions of interest for each time window (200–400 and 400–600 ms). For the N400 effect, a repeated-measures ANOVA with Condition (Overlapping vs. Unrelated) and Hemisphere (Left vs. Right) as within-subject factors and Group (Controls vs. Native signers) as a between-subject factor was conducted for the central regions of interest, for each time window (200–400 and 400–600 ms) separately.

In the 200–400 ms time window, regions of interest for the P350 effect revealed a significant four-way interaction of Condition × Hemisphere × Region × Group [*F*(1,26) = 8.41, *p* = 0.008], which we followed up by separate repeated-measures ANOVAs per region. For anterior regions of interest, no significant main effects of Condition, Hemisphere or Group nor any significant interactions between factors were found (all *p* > 0.29). For posterior regions of interest, significant main effects of Condition [*F*(1,26) = 38.06, *p* < 0.001] and Hemisphere [*F*(1,26) = 6.68, *p* = 0.016] were modulated by a significant Condition × Hemisphere interaction [*F*(1,26) = 5.10, *p* = 0.033]. *Post hoc* comparisons with Tukey’s correction to account for multiple comparisons showed that mean amplitude in the 200–400 ms time window was more positive in the Unrelated condition compared to the Overlapping condition in the left posterior region [*M* = −3.01, *SE* = 0.50, *t*(51.9) = −6.02, *p* < 0.001] as well as in the right posterior region [*M* = −1.45, *SE* = 0.50, *t*(51.9) = −2.90, *p* = 0.027]. The main effect of Group was not significant [*F*(1,26) = 0.28, *p* = 0.60]; nor were any of the interactions with Group (all *p* > 0.08; for full results see Supplementary Tables 1A–D). Regions of interest for the N400 revealed a main effect of Condition [*F*(1,26) = 21.40, *p* < 0.001] only, with more negative amplitudes in the Unrelated condition than in the Overlapping condition. No other effects or interactions were significant (all *p* > 0.06, see Supplementary Tables 3, 4 for full results).

In the 400–600 ms time window, regions of interest for the P350 effect revealed a significant four-way interaction of Condition × Hemisphere × Region × Group [*F*(1,26) = 9.42, *p* = 0.005] which we followed up by separate repeated-measures ANOVAs per region. For anterior regions of interest, no significant main effects of Condition, Hemisphere or Group nor any significant interactions between factors were found (all *p* > 0.37). For posterior regions of interest, significant main effects of Condition [*F*(1,26) = 7.17, *p* = 0.013] and Hemisphere [*F*(1,26) = 6.13, *p* = 0.020] were modulated by a significant Condition × Hemisphere × Group interaction [*F*(1,26) = 5.74, *p* = 0.024]. The main effect of Group was not significant [*F*(1,26) = 0.90, *p* = 0.35], nor were other interactions with Group (all *p* > 0.07). *Post hoc* comparisons with Tukey’s correction to account for multiple comparisons showed that mean amplitude in the 400–600 ms time window was more positive in the Unrelated condition compared to the Overlapping condition in the right posterior region in Native signers only [*M* = −3.20, *SE* = 0.94, *t*(45.8) = −3.39, *p* = 0.028; all other *p* > 0.54; for full results see Supplementary Tables 2A–D]. Regions of interest for the N400 revealed a main effect of Condition [*F*(1,26) = 20.00, *p* < 0.001] only, with more negative amplitudes in the Unrelated condition than in the Overlapping condition (all *p* > 0.27, see Supplementary Tables 3, 4 for full results).

## Discussion

The main aim of the present study was to investigate whether and when during online processing beginning readers link signed language (deaf participants) or spoken language (hearing participants) to written word recognition. We tested two groups of children: congenitally deaf (native signing) and hearing beginning readers, who were matched on reading skill. Our behavioral results showed that both groups of children matched word onsets (signed or spoken) and written target words. Signed and spoken onsets facilitated lexical decisions to corresponding written words. ERPs were informative regarding aspects of processing that were involved when deaf and hearing children link their native language to reading. In both groups, rapid priming effects emerged as early as 200 ms after the onset of the written target word. That is, deaf and hearing beginning readers appeared to have used incremental processing in their native language for written word processing as rapidly as hearing adults do (see Friedrich et al., 2004a,b, 2008, 2013; Friedrich, 2005). Moreover, we found similar ERP deflections in onset priming in signing and hearing children and these ERP deflections resemble those in hearing adults. Together, our results suggest that the links attested in previous studies between sign language proficiency and reading in deaf readers (Padden and Ramsey, 1998; Chamberlain and Mayberry, 2008) might – at least in part – be mediated by implicit associations between representations of signs and written words that are automatically accessed already by beginning readers.

Facilitated lexical decision responses for targets which were preceded by a related word onset demonstrate that children link spoken language processing (hearing children) as well as sign language processing (deaf children) to reading. For hearing children, intimate links between spoken and written language are well established (for review see Goswami and Bryant, 2016). At the behavioral level, spoken-written priming of phonologically related words has been formerly observed for 8–10-year-olds (Clahsen and Fleischhauer, 2014; Quémart et al., 2018). For signing children, the present behavioral results are in line with findings showing links between sign language processing and reading in signing deaf adults (see Ormel and Giezen, 2014; Giezen and Emmorey, 2016 for reviews) and signing deaf children (Ormel et al., 2012; Villwock et al., 2021). However, response latencies reflect only the outcome of complex recognition and decision processes and do not allow disentangling whether facilitation reflects early, rather automatic, or later, rather decision related aspects of processing (for further discussion see Friedrich et al., 2013; Schild and Friedrich, 2018). In that respect, our neurocognitive data strengthen those claims. Moreover, they expand them and add more detailed information to those research questions.

Across both groups of children, ERP effects related to prime-target overlap manifested first in a time window ranging from 200 and 400 ms after target word onset. At lateral electrode leads, prime-target overlap elicited more positive amplitudes (the P350 effect), whereas, at central electrodes, prime-target overlap elicited more negative amplitudes (the N400 effect) compared to unrelated pairs, respectively. This is in line with P350 and N400 effects previously reported for spoken - written word onset priming with hearing adults (Friedrich et al., 2004a,b, 2008, 2013; Friedrich, 2005). In contrast to ERP effects found for spoken-written word onset priming in hearing adults, P350 difference topographies in hearing and signing children were more pronounced for posterior than for anterior electrode leads. Thus, topographies of P350 effects for written target words appear to follow a posterior to anterior gradient from middle childhood to adulthood. This is somewhat remarkable as spoken word onset priming elicited comparable P350 difference topographies with (left-)anterior distribution of the effect in hearing adults (e.g., Friedrich et al., 2009; Schild et al., 2014; Schild and Friedrich, 2018) and in hearing children (preschoolers and first graders; Schild et al., 2011, Schild et al., 2014b). That is, there was no posterior to anterior gradient of P350 effects for spoken targets during development. We might conclude that the neural processing of written words, as tapped by spoken-written word onset priming, undergoes more restructuring during development from middle childhood to adulthood than the neural processing of spoken words does. Nevertheless, we have to consider that we presented video clips of the speakers in the present study, while we presented unimodal spoken materials in all previous studies with children and adults.

In particular the earlier time window of ERP differences between related and unrelated pairs is associated with lexical access in written word recognition in hearing adults (e.g., Grainger et al., 2006; Holcomb and Grainger, 2006, 2007) and in signing adults (for signing readers see: Gutierrez-Sigut et al., 2017). Therefore, we conclude that hearing children are linking incremental processing in the spoken domain to written word processing as early as hearing adults do. Similarly, signing children are linking incremental processing of signs to written word processing as early as adult signers do. In particular, our results show that beginning readers are sensitive to the outcome of some sort of matching between representations of different modalities (spoken/signed and written) and target word processing is affected by mismatches. Note that this is the first study providing neurocognitive data with high temporal resolution demonstrating that deaf signing children rapidly exploit sign word onsets to facilitate written word identification. Our results point to the conclusion that native signing children are activating written word representations on the basis of sign word onsets similar to hearing children activating written word representations on the basis of spoken word onsets.

How could incrementally processed signs modulate the processing of written words? One possibility is that mouthings, which are relatively common in DGS, might provide some direct form hints between signs and written word representations. Mouthings relate to the phonology of spoken language as they are speech-derived mouth actions accompanying manual signs (Boyes-Braem and Sutton-Spence, 2001; Brentari, 2011). There might be some grapheme-mouthing correspondence between DGS and written German, which deaf native signers can use similarly to grapheme-phoneme correspondence that hearing readers use. Studies focusing on co-active mouth patterns in deaf readers report some reliable mapping between orthography and mouthing (Vinson et al., 2010; Giustolisi et al., 2017). This is consistent with an fMRI study demonstrating that mouthings accompanied by signs generated activations similar to speech reading, while mouth patterns unrelated to spoken language (called “mouth gestures”) generated activations similar to manual signs without mouth movements (Capek et al., 2008). Indeed, we found naturally produced mouth patterns in the sign word onsets that we presented (see video examples in the Supplementary Material). Therefore, our results might further inform the ongoing debate on whether mouthings and manual components have shared lexical representations or whether mouthings occur incidentally by simultaneous code mixing and blending (see Sutton-Spence, 1999; Boyes-Braem and Sutton-Spence, 2001; Sutton-Spence and Day, 2001; Emmorey et al., 2005; Johnston and Schembri, 2007; Nadolske and Rosenstock, 2007; Donati and Branchini, 2013).

In particular the early onset of effects in the ERPs obtained for both groups of beginning readers might confirm the assumption that lexical access is neither selective to the modality (sign, spoken, or written) nor to the language (sign language or written language) of the input that the system receives (Morford et al., 2011, 2019). Non-selective lexical access is well established in research with hearing bilinguals (Spivey and Marian, 1999; Lagrou et al., 2011; for review see Kroll et al., 2015). In previous spoken-written word onset priming studies with hearing adults, we already related the P350 effect to modality-independent lexical access (Friedrich et al., 2004a,^2013^; Friedrich, 2005). The present results suggest that the ERP effects obtained in word onset priming might be language non-selective as well. With respect to native signing beginning readers, we might conclude that they can facilitate lexical access to written word recognition via an implicit language non-selective linguistic pathway (for a similar conclusion drawn from priming data see Villwock et al., 2021). Hence, reading proficiency might well be modulated by sign language proficiency (McQuarrie and Abbott, 2013; Corina et al., 2014; Holmer et al., 2016). Moreover, deaf readers might even uniquely benefit from language non-selective lexical access during reading since there is typically less competition between phonological and orthographical patterns between signs and words compared to competition between co-activated spoken words and respective written words in hearing individuals (for further discussion see Morford et al., 2019).

P350 effects elicited in signing and hearing beginning readers were similar in their timing, but differed in the lateralization of the posteriorly distributed ERP differences. Similar to previous spoken-written priming studies with adults (Friedrich et al., 2004a,b, 2008, 2009), hearing children in the present study showed a left-lateralized P350 effect. In contrast, deaf native signing children showed a bilateral distribution of the P350 effect. Given the inverse problem in ERP research, topographic ERP effects have to be interpreted with caution. However, the different topography of P350 effects in hearing and deaf beginning readers that we obtained here is consistent with topographic differences of ERP effects formerly shown for deaf and reading adults (Neville et al., 1982; Emmorey et al., 2017; Sehyr et al., 2020). For example, for word reading, individual reading ability was associated with a larger N170 over right-hemispheric occipital sites for deaf readers. By contrast, reading ability was associated with a smaller N170 over the right hemisphere for hearing readers (Emmorey et al., 2017; Sehyr et al., 2020). These ERP findings converge with fMRI evidence for more bilaterally distributed networks that deaf signers recruit for reading (compared to hearing readers; Emmorey et al., 2013). In light of these ERP and fMRI studies with deaf adult readers, the bilateral P350 response in deaf native signing children (compared to the left-lateralized response in reading matched hearing children) might integrate into the assumption that deaf signers recruit the right hemisphere for processing visual word forms to a greater extent than hearing readers do (see also Emmorey and Lee, 2021).

Following the P350 effect and the N400 effect in the ERPs, there was evidence for some later ERP differences between overlapping and unrelated prime-target pairs (see Figure 5). Formerly, we discussed extended positive-going ERP effects for related spoken word targets as being evidence for long-lasting facilitation of respective candidate words (see Friedrich et al., 2013). However, the present design does not allow us to compare these extended ERP effects to the processing of more or less appropriate related candidate words as we did in the former study with matching and partially mismatching spoken target words. In addition to extended facilitation, late ERP effects might also reflect strategic effects associated with the lexical decision responses, which ranged between approximately 820 and 1,000 ms after target word onset (see also Friedrich et al., 2013). Future research with systematically varied prime-target overlap has to further investigate the functional role of these late ERP effects, which appear to be more pronounced in the beginning signing readers than in the beginning hearing readers (see Figure 5).

With regard to different sub-processes that might be reflected in the P350 effect, in the N400 effect and in the reaction times (for further discussion see Friedrich et al., 2013; Schild and Friedrich, 2018), word onset priming might provide a promising tool to further investigate aspects of processing that might have contributed to diverging ERP effects obtained in previous sign priming studies. For example, signed prime-target pairs overlapping in sign units have been found to either cause behavioral facilitation (Dye and Shih, 2006), no effect (Mayberry and Witcher, 2005), or even inhibition (Corina and Hildebrandt, 2002; Carreiras et al., 2008; compared to unrelated prime-target pairs, respectively). In parallel, some ERP studies have revealed either a reduced N400 when signed primes and targets overlapped (compared to completely unrelated prime-target pairs; for ASL: Meade et al., 2018; Hosemann et al., 2020), while others obtained an enhanced N400 for overlap (compared to completely unrelated prime-target pairs; Baus and Carreiras, 2012; Gutierrez et al., 2012a). This might suggest that different sign parameters differently affect different aspects of processing. A recent eye tracking study on Cantonese reading with Hong Kong Sign Language systematically varying different sign parameters also pointed in that direction (Thierfelder et al., 2020). One might suggest that the parallel activation of multiple memory representations for words (presumably reflected in the P350 effect) and the selection of the most promising candidate among them (presumably reflected in the N400 effect and in the lexical decision latencies) are differently sensitive to different units of sign language.

## Conclusion

The present study indicates that deaf beginning readers engage rapid sign language co-activation during visual word recognition. Sign onset primes modulated ERP responses of following written target words with lexical overlap to the primes. ERP effects started 200 ms after target word onset. This suggests that deaf beginning readers implicitly link signs to written word recognition. In addition, consecutive selection mechanisms underlying the behavioral responses appeared to be facilitated for matching targets. Our results demonstrate co-activation from DGS as a native language to written German as a second language in deaf signing beginning readers. ERPs recorded in signing individuals might be a promising tool to disentangle which incremental and lexical processes in written word recognition link to which aspects of processing in the signed domain.

## Data Availability Statement

The raw data supporting the conclusions of this article will be made available by the authors, without undue reservation.

## Ethics Statement

The studies involving human participants were reviewed and approved by the Ethikkommission der Deutschen Gesellschaft für Psychologie. Written informed consent to participate in this study was provided by the participants’ legal guardian/next of kin. Written informed consent was obtained from the individual(s) for the publication of any potentially identifiable images or data included in this article.

## Author Contributions

BH-F: conceptualization, funding, supervision, investigation, writing – original draft, and writing – review and editing. MG: formal analysis, investigation, and writing – review and editing. BR: conceptualization, funding, supervision, and writing –review and editing. CF: conceptualization, funding, supervision, investigation, and writing – reviewing and editing. All authors contributed to the article and approved the submitted version.

## Conflict of Interest

The authors declare that the research was conducted in the absence of any commercial or financial relationships that could be construed as a potential conflict of interest.

## Publisher’s Note

All claims expressed in this article are solely those of the authors and do not necessarily represent those of their affiliated organizations, or those of the publisher, the editors and the reviewers. Any product that may be evaluated in this article, or claim that may be made by its manufacturer, is not guaranteed or endorsed by the publisher.

## Footnotes

1 Sign fragments such as “KU” for KUCHEN (cake) or “WE” for WECKER (alarm clock) are sign onsets with a very brief movement until the hands are in the correct position (*location)* of the intended sign (see section “Stimuli”).

2
www.neurobs.com
