# Supplementary material for: Ongoing Sign Processing Facilitates Written Word Recognition in Deaf Native Signing Children
Source: Front Psychol. 2022 Aug 5;13:917700. doi: 10.3389/fpsyg.2022.917700 (PMC9390089; doi:10.3389/fpsyg.2022.917700)
Supplement: Supplementary file 1 [file Data_Sheet_1.docx]

**Table 1a: Positivity/P350 - Repeated measures ANOVA for the mean amplitude in the 200-400 ms time window**

| Within Subjects Effects | | | | | | | | | | | | | | | |
| --- | --- | --- | --- | --- | --- | --- | --- | --- | --- | --- | --- | --- | --- | --- | --- |
|  |  |  |  |  |  |  |  |  |  |  |  |  |  |  |  |
|  | | **Sum of Squares** | | **df** | | **Mean Square** | | ***F*** | | ***p*** | | **η²** | | **partial η²** | |
| Condition |  | 82.2354 |  | 1 |  | 82.2354 |  | 22.01455 |  | < .001 |  | 0.019 |  | 0.458 |  |
| Condition ✻ Group |  | 7.2414 |  | 1 |  | 7.2414 |  | 1.93854 |  | 0.176 |  | 0.002 |  | 0.069 |  |
| Residual |  | 97.1231 |  | 26 |  | 3.7355 |  |  |  |  |  |  |  |  |  |
| Hemisphere |  | 64.9830 |  | 1 |  | 64.9830 |  | 3.19966 |  | 0.085 |  | 0.015 |  | 0.110 |  |
| Hemisphere ✻ Group |  | 24.8225 |  | 1 |  | 24.8225 |  | 1.22222 |  | 0.279 |  | 0.006 |  | 0.045 |  |
| Residual |  | 528.0434 |  | 26 |  | 20.3094 |  |  |  |  |  |  |  |  |  |
| Region |  | 703.3316 |  | 1 |  | 703.3316 |  | 11.40196 |  | 0.002 |  | 0.164 |  | 0.305 |  |
| Region ✻ Group |  | 7.7894 |  | 1 |  | 7.7894 |  | 0.12628 |  | 0.725 |  | 0.002 |  | 0.005 |  |
| Residual |  | 1603.8144 |  | 26 |  | 61.6852 |  |  |  |  |  |  |  |  |  |
| Condition ✻ Hemisphere |  | 10.3763 |  | 1 |  | 10.3763 |  | 2.33778 |  | 0.138 |  | 0.002 |  | 0.082 |  |
| Condition ✻ Hemisphere ✻ Group |  | 2.7141 |  | 1 |  | 2.7141 |  | 0.61149 |  | 0.441 |  | 0.001 |  | 0.023 |  |
| Residual |  | 115.4023 |  | 26 |  | 4.4386 |  |  |  |  |  |  |  |  |  |
| Condition ✻ Region |  | 58.0596 |  | 1 |  | 58.0596 |  | 21.06781 |  | < .001 |  | 0.014 |  | 0.448 |  |
| Condition ✻ Region ✻ Group |  | 0.0169 |  | 1 |  | 0.0169 |  | 0.00613 |  | 0.938 |  | 0.000 |  | 0.000 |  |
| Residual |  | 71.6520 |  | 26 |  | 2.7558 |  |  |  |  |  |  |  |  |  |
| Hemisphere ✻ Region |  | 80.0495 |  | 1 |  | 80.0495 |  | 9.59040 |  | 0.005 |  | 0.019 |  | 0.269 |  |
| Hemisphere ✻ Region ✻ Group |  | 19.5013 |  | 1 |  | 19.5013 |  | 2.33637 |  | 0.138 |  | 0.005 |  | 0.082 |  |
| Residual |  | 217.0177 |  | 26 |  | 8.3468 |  |  |  |  |  |  |  |  |  |
|  |  |  |  |  |  |  |  |  |  |  |  |  |  |  |  |
| Condition ✻ Hemisphere ✻ Region |  | 6.8059 |  | 1 |  | 6.8059 |  | 5.81196 |  | 0.023 |  | 0.002 |  | 0.183 |  |
| Condition ✻ Hemisphere ✻ Region ✻ Group |  | 9.8444 |  | 1 |  | 9.8444 |  | 8.40677 |  | 0.008 |  | 0.002 |  | 0.244 |  |
| Residual |  | 30.4462 |  | 26 |  | 1.1710 |  |  |  |  |  |  |  |  |  |
| Note. Type 3 Sums of Squares | | | | | | | | | | | | | | | |
|  | | | | | | | | | | | | | | | |

| Between Subjects Effects | | | | | | | | | | | | | | | |
| --- | --- | --- | --- | --- | --- | --- | --- | --- | --- | --- | --- | --- | --- | --- | --- |
|  |  |  |  |  |  |  |  |  |  |  |  |  |  |  |  |
|  | | **Sum of Squares** | | **df** | | **Mean Square** | | ***F*** | | ***p*** | | **η²** | | **partial η²** | |
| Group |  | 7.73 |  | 1 |  | 7.73 |  | 0.376 |  | 0.545 |  | 0.002 |  | 0.014 |  |
| Residual |  | 534.40 |  | 26 |  | 20.55 |  |  |  |  |  |  |  |  |  |
| Note. Type 3 Sums of Squares | | | | | | | | | | | | | | | |
|  | | | | | | | | | | | | | | | |

**Table 1b: Positivity/P350 - Repeated measures ANOVA for the mean amplitude in the 200-400 ms time window in anterior regions**

| Within Subjects Effects | | | | | | | | | | | | | | | | | | | | | | | | | | | | | | | |
| --- | --- | --- | --- | --- | --- | --- | --- | --- | --- | --- | --- | --- | --- | --- | --- | --- | --- | --- | --- | --- | --- | --- | --- | --- | --- | --- | --- | --- | --- | --- | --- |
|  | | | | |  | |  | | |  | |  |  |  | | | | |  |  | | |  |  | | |  |  |  |  |  |
|  | | | | | | | **Sum of Squares** | | | | | **df** | | **Mean Square** | | | | | | ***F*** | | | | ***p*** | | | | **η²** | | **partial η²** | |
| Condition | | | | |  | | 1.049 | | |  | | 1 |  | 1.049 | | | | |  | 0.3705 | | |  | 0.548 | | |  | 0.001 |  | 0.014 |  |
| Condition ✻ Group | | | | |  | | 3.279 | | |  | | 1 |  | 3.279 | | | | |  | 1.1578 | | |  | 0.292 | | |  | 0.003 |  | 0.043 |  |
| Residual | | | | |  | | 73.646 | | |  | | 26 |  | 2.833 | | | | |  |  | | |  |  | | |  |  |  |  |  |
| Hemisphere | | | | |  | | 0.392 | | |  | | 1 |  | 0.392 | | | | |  | 0.0561 | | |  | 0.815 | | |  | 0.000 |  | 0.002 |  |
| Hemisphere ✻ Group | | | | |  | | 0.160 | | |  | | 1 |  | 0.160 | | | | |  | 0.0229 | | |  | 0.881 | | |  | 0.000 |  | 0.001 |  |
| Residual | | | | |  | | 181.716 | | |  | | 26 |  | 6.989 | | | | |  |  | | |  |  | | |  |  |  |  |  |
| Condition ✻ Hemisphere | | | | |  | | 0.188 | | |  | | 1 |  | 0.188 | | | | |  | 0.0824 | | |  | 0.776 | | |  | 0.000 |  | 0.003 |  |
| Condition ✻ Hemisphere ✻ Group | | | | |  | | 1.110 | | |  | | 1 |  | 1.110 | | | | |  | 0.4876 | | |  | 0.491 | | |  | 0.001 |  | 0.018 |  |
| Residual | | | | |  | | 59.194 | | |  | | 26 |  | 2.277 | | | | |  |  | | |  |  | | |  |  |  |  |  |
| Note. Type 3 Sums of Squares | | | | | | | | | | | | | | | | | | | | | | | | | | | | | | | |
|  | | | | | | | | | | | | | | | | | | | | | | | | | | | | | | | |
| Between Subjects Effects | | | | | | | | | | | | | | | | | | | | | | | | | |  |  |  |  |  |  |
|  |  |  |  |  | |  | |  |  | |  | | | |  |  |  |  | | |  |  | | |  |  |  |  |  |  |  |
|  | | **Sum of Squares** | | **df** | | | | **Mean Square** | | | ***F*** | | | | | ***p*** | | **η²** | | | | **partial η²** | | | |  |  |  |  |  |  |
| Group |  | 6.15e-5 |  | 1 | |  | | 6.15e-5 |  | | 2.26e-6 | | | |  | 0.999 |  | 0.000 | | |  | 0.000 | | |  |  |  |  |  |  |  |
| Residual |  | 709 |  | 26 | |  | | 27.3 |  | |  | | | |  |  |  |  | | |  |  | | |  |  |  |  |  |  |  |
| Note. Type 3 Sums of Squares | | | | | | | | | | | | | | | | | | | | | | | | | |  |  |  |  |  |  |
|  | | | | | | | | | | | | | | | | | | | | | | | | | |  |  |  |  |  |  |

# Table 1c: Positivity/P350 - Repeated measures ANOVA for the mean amplitude in the 200-400 ms time window in posterior regions

| Within Subjects Effects | | | | | | | | | | | | | | | | | | | | | | | | | | | | | | |
| --- | --- | --- | --- | --- | --- | --- | --- | --- | --- | --- | --- | --- | --- | --- | --- | --- | --- | --- | --- | --- | --- | --- | --- | --- | --- | --- | --- | --- | --- | --- |
|  | | | | | |  | |  | |  |  | |  | |  | | |  | |  | |  |  | | |  |  |  |  |  |
|  | | | | | | | | **Sum of Squares** | | | **df** | | | | **Mean Square** | | | | | ***F*** | | | ***p*** | | | | **η²** | | **partial η²** | |
| Condition | | | | | |  | | 139.25 | |  | 1 | |  | | 139.25 | | |  | | 38.06 | |  | < .001 | | |  | 0.055 |  | 0.594 |  |
| Condition ✻ Group | | | | | |  | | 3.98 | |  | 1 | |  | | 3.98 | | |  | | 1.09 | |  | 0.307 | | |  | 0.002 |  | 0.040 |  |
| Residual | | | | | |  | | 95.13 | |  | 26 | |  | | 3.66 | | |  | |  | |  |  | | |  |  |  |  |  |
| Hemisphere | | | | | |  | | 144.64 | |  | 1 | |  | | 144.64 | | |  | | 6.68 | |  | 0.016 | | |  | 0.057 |  | 0.204 |  |
| Hemisphere ✻ Group | | | | | |  | | 44.16 | |  | 1 | |  | | 44.16 | | |  | | 2.04 | |  | 0.165 | | |  | 0.017 |  | 0.073 |  |
| Residual | | | | | |  | | 563.35 | |  | 26 | |  | | 21.67 | | |  | |  | |  |  | | |  |  |  |  |  |
| Condition ✻ Hemisphere | | | | | |  | | 16.99 | |  | 1 | |  | | 16.99 | | |  | | 5.10 | |  | 0.033 | | |  | 0.007 |  | 0.164 |  |
| Condition ✻ Hemisphere ✻ Group | | | | | |  | | 11.45 | |  | 1 | |  | | 11.45 | | |  | | 3.43 | |  | 0.075 | | |  | 0.004 |  | 0.117 |  |
| Residual | | | | | |  | | 86.65 | |  | 26 | |  | | 3.33 | | |  | |  | |  |  | | |  |  |  |  |  |
| Note. Type 3 Sums of Squares | | | | | | | | | | | | | | | | | | | | | | | | | | | | | | |
|  | | | | | | | | | | | | | | | | | | | | | | | | | | | | | | |
| Between Subjects Effects | | | | | | | | | | | | | | | | | | | | | | | | | | | | | | |
|  |  |  |  |  |  | |  | |  |  | |  | |  | |  |  | |  | |  | | |  |  |  |  |  |  |  |
|  | | **Sum of Squares** | | **df** | | | **Mean Square** | | | ***F*** | | | | ***p*** | | | **η²** | | | | **partial η²** | | | |  |  |  |  |  |  |
| Group |  | 15.5 |  | 1 |  | | 15.5 | |  | 0.282 | |  | | 0.600 | |  | 0.006 | |  | | 0.011 | | |  |  |  |  |  |  |  |
| Residual |  | 1429.3 |  | 26 |  | | 55.0 | |  |  | |  | |  | |  |  | |  | |  | | |  |  |  |  |  |  |  |
| Note. Type 3 Sums of Squares | | | | | | | | | | | | | | | | | | | | | | | | |  |  |  |  |  |  |

**Table 1d: Positivity/P350 – Post hoc comparisons for the mean amplitude in the 200-400 ms time window in posterior regions**

|  | | | | | | | | | | | | | | | | | | | |
| --- | --- | --- | --- | --- | --- | --- | --- | --- | --- | --- | --- | --- | --- | --- | --- | --- | --- | --- | --- |
| **Comparison** | | | | | | | | | |  | | | | | | | | | |
| **Condition** | | **Hemisphere** | |  | | **Condition** | | **Hemisphere** | | **Mean Difference** | | **SE** | | **df** | | ***t*** | | ***p_tukey_*** | |
| Overlapping |  | Left |  | - |  | Overlapping |  | Right |  | -3.0519 |  | 0.945 |  | 33.8 |  | -3.2298 |  | 0.014 |  |
|  |  |  |  | - |  | Unrelated |  | Left |  | -3.0091 |  | 0.500 |  | 51.9 |  | -6.0218 |  | < .001 |  |
|  |  |  |  | - |  | Unrelated |  | Right |  | -4.5029 |  | 0.951 |  | 34.5 |  | -4.7346 |  | < .001 |  |
|  |  | Right |  | - |  | Unrelated |  | Left |  | 0.0428 |  | 0.951 |  | 34.5 |  | 0.0450 |  | 1.000 |  |
|  |  |  |  | - |  | Unrelated |  | Right |  | -1.4510 |  | 0.500 |  | 51.9 |  | -2.9037 |  | 0.027 |  |
| Unrelated |  | Left |  | - |  | Unrelated |  | Right |  | -1.4937 |  | 0.945 |  | 33.8 |  | -1.5808 |  | 0.403 |  |
|  | | | | | | | | | | | | | | | | | | | |

# Table 2a: Positivity/P350 - Repeated measures ANOVA for the mean amplitude in the 400-600 ms time window

| Within Subjects Effects | | | | | | | | | | | | | | | |
| --- | --- | --- | --- | --- | --- | --- | --- | --- | --- | --- | --- | --- | --- | --- | --- |
|  |  |  |  |  |  |  |  |  |  |  |  |  |  |  |  |
|  | | **Sum of Squares** | | **df** | | **Mean Square** | | ***F*** | | ***p*** | | **η²** | | **partial η²** | |
| Condition |  | 35.776 |  | 1 |  | 35.776 |  | 5.0757 |  | 0.033 |  | 0.006 |  | 0.163 |  |
| Condition ✻ Group |  | 7.692 |  | 1 |  | 7.692 |  | 1.0913 |  | 0.306 |  | 0.001 |  | 0.040 |  |
| Residual |  | 183.260 |  | 26 |  | 7.048 |  |  |  |  |  |  |  |  |  |
| Hemisphere |  | 93.480 |  | 1 |  | 93.480 |  | 3.4355 |  | 0.075 |  | 0.015 |  | 0.117 |  |
| Hemisphere ✻ Group |  | 0.661 |  | 1 |  | 0.661 |  | 0.0243 |  | 0.877 |  | 0.000 |  | 0.001 |  |
| Residual |  | 707.461 |  | 26 |  | 27.210 |  |  |  |  |  |  |  |  |  |
| Region |  | 2062.888 |  | 1 |  | 2062.888 |  | 32.9071 |  | < .001 |  | 0.331 |  | 0.559 |  |
| Region ✻ Group |  | 117.778 |  | 1 |  | 117.778 |  | 1.8788 |  | 0.182 |  | 0.019 |  | 0.067 |  |
| Residual |  | 1629.893 |  | 26 |  | 62.688 |  |  |  |  |  |  |  |  |  |
| Condition ✻ Hemisphere |  | 0.448 |  | 1 |  | 0.448 |  | 0.0930 |  | 0.763 |  | 0.000 |  | 0.004 |  |
| Condition ✻ Hemisphere ✻ Group |  | 5.250 |  | 1 |  | 5.250 |  | 1.0906 |  | 0.306 |  | 0.001 |  | 0.040 |  |
| Residual |  | 125.151 |  | 26 |  | 4.814 |  |  |  |  |  |  |  |  |  |
| Condition ✻ Region |  | 25.446 |  | 1 |  | 25.446 |  | 5.9181 |  | 0.022 |  | 0.004 |  | 0.185 |  |
| Condition ✻ Region ✻ Group |  | 24.106 |  | 1 |  | 24.106 |  | 5.6065 |  | 0.026 |  | 0.004 |  | 0.177 |  |
| Residual |  | 111.792 |  | 26 |  | 4.300 |  |  |  |  |  |  |  |  |  |
| Hemisphere ✻ Region |  | 65.840 |  | 1 |  | 65.840 |  | 5.5867 |  | 0.026 |  | 0.011 |  | 0.177 |  |
| Hemisphere ✻ Region ✻ Group |  | 14.943 |  | 1 |  | 14.943 |  | 1.2679 |  | 0.270 |  | 0.002 |  | 0.046 |  |
| Residual |  | 306.416 |  | 26 |  | 11.785 |  |  |  |  |  |  |  |  |  |
| Condition ✻ Hemisphere ✻ Region |  | 0.709 |  | 1 |  | 0.709 |  | 0.3405 |  | 0.565 |  | 0.000 |  | 0.013 |  |
| Condition ✻ Hemisphere ✻ Region ✻ Group |  | 19.622 |  | 1 |  | 19.622 |  | 9.4248 |  | 0.005 |  | 0.003 |  | 0.266 |  |
| Residual |  | 54.130 |  | 26 |  | 2.082 |  |  |  |  |  |  |  |  |  |
| Note. Type 3 Sums of Squares | | | | | | | | | | | | | | | |
|  | | | | | | | | | | | | | | | |

| Between Subjects Effects | | | | | | | | | | | | | | | |
| --- | --- | --- | --- | --- | --- | --- | --- | --- | --- | --- | --- | --- | --- | --- | --- |
|  |  |  |  |  |  |  |  |  |  |  |  |  |  |  |  |
|  | | **Sum of Squares** | | **df** | | **Mean Square** | | ***F*** | | ***p*** | | **η²** | | **partial η²** | |
| Group |  | 0.568 |  | 1 |  | 0.568 |  | 0.0230 |  | 0.881 |  | 0.000 |  | 0.001 |  |
| Residual |  | 642.512 |  | 26 |  | 24.712 |  |  |  |  |  |  |  |  |  |
| Note. Type 3 Sums of Squares | | | | | | | | | | | | | | | |
|  | | | | | | | | | | | | | | | |

**Table 2b: Positivity/P350 - Repeated measures ANOVA for the mean amplitude in the 400-600 ms time window in anterior regions**

| Within Subjects Effects | | | | | | | | | | | | | | | |
| --- | --- | --- | --- | --- | --- | --- | --- | --- | --- | --- | --- | --- | --- | --- | --- |
|  |  |  |  |  |  |  |  |  |  |  |  |  |  |  |  |
|  | | **Sum of Squares** | | **df** | | **Mean Square** | | ***F*** | | ***p*** | | **η²** | | **partial η²** | |
| Condition |  | 0.4389 |  | 1 |  | 0.4389 |  | 0.15297 |  | 0.699 |  | 0.000 |  | 0.006 |  |
| Condition ✻ Group |  | 2.2820 |  | 1 |  | 2.2820 |  | 0.79540 |  | 0.381 |  | 0.002 |  | 0.030 |  |
| Residual |  | 74.5935 |  | 26 |  | 2.8690 |  |  |  |  |  |  |  |  |  |
| Hemisphere |  | 1.2079 |  | 1 |  | 1.2079 |  | 0.09150 |  | 0.765 |  | 0.001 |  | 0.004 |  |
| Hemisphere ✻ Group |  | 10.9456 |  | 1 |  | 10.9456 |  | 0.82911 |  | 0.371 |  | 0.008 |  | 0.031 |  |
| Residual |  | 343.2442 |  | 26 |  | 13.2017 |  |  |  |  |  |  |  |  |  |
| Condition ✻ Hemisphere |  | 0.0150 |  | 1 |  | 0.0150 |  | 0.00505 |  | 0.944 |  | 0.000 |  | 0.000 |  |
| Condition ✻ Hemisphere ✻ Group |  | 2.2865 |  | 1 |  | 2.2865 |  | 0.77164 |  | 0.388 |  | 0.002 |  | 0.029 |  |
| Residual |  | 77.0421 |  | 26 |  | 2.9632 |  |  |  |  |  |  |  |  |  |
| Note. Type 3 Sums of Squares | | | | | | | | | | | | | | | |
|  | | | | | | | | | | | | | | | |

| Between Subjects Effects | | | | | | | | | | | | | | | |
| --- | --- | --- | --- | --- | --- | --- | --- | --- | --- | --- | --- | --- | --- | --- | --- |
|  |  |  |  |  |  |  |  |  |  |  |  |  |  |  |  |
|  | | **Sum of Squares** | | **df** | | **Mean Square** | | ***F*** | | ***p*** | | **η²** | | **partial η²** | |
| Group |  | 67.3 |  | 1 |  | 67.3 |  | 2.18 |  | 0.152 |  | 0.049 |  | 0.077 |  |
| Residual |  | 803.4 |  | 26 |  | 30.9 |  |  |  |  |  |  |  |  |  |
| Note. Type 3 Sums of Squares | | | | | | | | | | | | | | | |

**Table 2c: Positivity/P350 - Repeated measures ANOVA for the mean amplitude in the 400-600 ms time window in posterior regions**

| Within Subjects Effects | | | | | | | | | | | | | | | |
| --- | --- | --- | --- | --- | --- | --- | --- | --- | --- | --- | --- | --- | --- | --- | --- |
|  |  |  |  |  |  |  |  |  |  |  |  |  |  |  |  |
|  | | **Sum of Squares** | | **df** | | **Mean Square** | | ***F*** | | ***p*** | | **η²** | | **partial η²** | |
| Condition |  | 60.78 |  | 1 |  | 60.78 |  | 7.168 |  | 0.013 |  | 0.022 |  | 0.216 |  |
| Condition ✻ Group |  | 29.52 |  | 1 |  | 29.52 |  | 3.481 |  | 0.073 |  | 0.011 |  | 0.118 |  |
| Residual |  | 220.46 |  | 26 |  | 8.48 |  |  |  |  |  |  |  |  |  |
| Hemisphere |  | 158.11 |  | 1 |  | 158.11 |  | 6.130 |  | 0.020 |  | 0.057 |  | 0.191 |  |
| Hemisphere ✻ Group |  | 4.66 |  | 1 |  | 4.66 |  | 0.181 |  | 0.674 |  | 0.002 |  | 0.007 |  |
| Residual |  | 670.63 |  | 26 |  | 25.79 |  |  |  |  |  |  |  |  |  |
| Condition ✻ Hemisphere |  | 1.14 |  | 1 |  | 1.14 |  | 0.290 |  | 0.595 |  | 0.000 |  | 0.011 |  |
| Condition ✻ Hemisphere ✻ Group |  | 22.58 |  | 1 |  | 22.58 |  | 5.743 |  | 0.024 |  | 0.008 |  | 0.181 |  |
| Residual |  | 102.24 |  | 26 |  | 3.93 |  |  |  |  |  |  |  |  |  |
| Note. Type 3 Sums of Squares | | | | | | | | | | | | | | | |

| Between Subjects Effects | | | | | | | | | | | | | | | |
| --- | --- | --- | --- | --- | --- | --- | --- | --- | --- | --- | --- | --- | --- | --- | --- |
|  |  |  |  |  |  |  |  |  |  |  |  |  |  |  |  |
|  | | **Sum of Squares** | | **df** | | **Mean Square** | | **F** | | **p** | | **η²** | | **partial η²** | |
| Group |  | 51.0 |  | 1 |  | 51.0 |  | 0.903 |  | 0.351 |  | 0.018 |  | 0.034 |  |
| Residual |  | 1469.0 |  | 26 |  | 56.5 |  |  |  |  |  |  |  |  |  |
| Note. Type 3 Sums of Squares | | | | | | | | | | | | | | | |

**Table 2d: Positivity/P350 – Post hoc comparisons for the mean amplitude in the 400-600 ms time window in posterior regions**

|  | | | | | | | | | | | | | | | | | | | | | | | |
| --- | --- | --- | --- | --- | --- | --- | --- | --- | --- | --- | --- | --- | --- | --- | --- | --- | --- | --- | --- | --- | --- | --- | --- |
| **Comparison** | | | | | | | | | | | | | |  | | | | | | | | | |
| **Condition** | | **Hemisphere** | | **group** | |  | | **Condition** | | **Hemisphere** | | **group** | | **Mean Difference** | | **SE** | | **df** | | ***t*** | | ***p_tukey_*** | |
| Overlapping |  | Left |  | Controls |  | - |  | Overlapping |  | Left |  | Native Signers |  | 1.070 |  | 1.839 |  | 59.1 |  | 0.5819 |  | 0.999 |  |
|  |  |  |  |  |  | - |  | Overlapping |  | Right |  | Controls |  | -3.884 |  | 1.457 |  | 33.7 |  | -2.6657 |  | 0.169 |  |
|  |  |  |  |  |  | - |  | Overlapping |  | Right |  | Native Signers |  | -0.202 |  | 1.839 |  | 59.1 |  | -0.1098 |  | 1.000 |  |
|  |  |  |  |  |  | - |  | Unrelated |  | Left |  | Controls |  | -1.547 |  | 0.942 |  | 45.8 |  | -1.6427 |  | 0.722 |  |
|  |  |  |  |  |  | - |  | Unrelated |  | Left |  | Native Signers |  | -0.734 |  | 1.839 |  | 59.1 |  | -0.3989 |  | 1.000 |  |
|  |  |  |  |  |  | - |  | Unrelated |  | Right |  | Controls |  | -3.231 |  | 1.565 |  | 41.4 |  | -2.0649 |  | 0.453 |  |
|  |  |  |  |  |  | - |  | Unrelated |  | Right |  | Native Signers |  | -3.398 |  | 1.839 |  | 59.1 |  | -1.8477 |  | 0.591 |  |
|  |  |  |  | Native Signers |  | - |  | Overlapping |  | Right |  | Controls |  | -4.955 |  | 1.839 |  | 59.1 |  | -2.6940 |  | 0.145 |  |
|  |  |  |  |  |  | - |  | Overlapping |  | Right |  | Native Signers |  | -1.272 |  | 1.457 |  | 33.7 |  | -0.8731 |  | 0.987 |  |
|  |  |  |  |  |  | - |  | Unrelated |  | Left |  | Controls |  | -2.617 |  | 1.839 |  | 59.1 |  | -1.4229 |  | 0.843 |  |
|  |  |  |  |  |  | - |  | Unrelated |  | Left |  | Native Signers |  | -1.804 |  | 0.942 |  | 45.8 |  | -1.9159 |  | 0.548 |  |
|  |  |  |  |  |  | - |  | Unrelated |  | Right |  | Controls |  | -4.301 |  | 1.839 |  | 59.1 |  | -2.3387 |  | 0.291 |  |
|  |  |  |  |  |  | - |  | Unrelated |  | Right |  | Native Signers |  | -4.468 |  | 1.565 |  | 41.4 |  | -2.8560 |  | 0.109 |  |
|  |  | Right |  | Controls |  | - |  | Overlapping |  | Right |  | Native Signers |  | 3.682 |  | 1.839 |  | 59.1 |  | 2.0022 |  | 0.489 |  |
|  |  |  |  |  |  | - |  | Unrelated |  | Left |  | Controls |  | 2.338 |  | 1.565 |  | 41.4 |  | 1.4940 |  | 0.806 |  |
|  |  |  |  |  |  | - |  | Unrelated |  | Left |  | Native Signers |  | 3.151 |  | 1.839 |  | 59.1 |  | 1.7131 |  | 0.679 |  |
|  |  |  |  |  |  | - |  | Unrelated |  | Right |  | Controls |  | 0.653 |  | 0.942 |  | 45.8 |  | 0.6939 |  | 0.997 |  |
|  |  |  |  |  |  | - |  | Unrelated |  | Right |  | Native Signers |  | 0.486 |  | 1.839 |  | 59.1 |  | 0.2643 |  | 1.000 |  |
|  |  |  |  | Native Signers |  | - |  | Unrelated |  | Left |  | Controls |  | -1.345 |  | 1.839 |  | 59.1 |  | -0.7312 |  | 0.996 |  |
|  |  |  |  |  |  | - |  | Unrelated |  | Left |  | Native Signers |  | -0.532 |  | 1.565 |  | 41.4 |  | -0.3398 |  | 1.000 |  |
|  |  |  |  |  |  | - |  | Unrelated |  | Right |  | Controls |  | -3.029 |  | 1.839 |  | 59.1 |  | -1.6469 |  | 0.720 |  |
|  |  |  |  |  |  | - |  | Unrelated |  | Right |  | Native Signers |  | -3.196 |  | 0.942 |  | 45.8 |  | -3.3946 |  | 0.028 |  |
| Unrelated |  | Left |  | Controls |  | - |  | Unrelated |  | Left |  | Native Signers |  | 0.813 |  | 1.839 |  | 59.1 |  | 0.4421 |  | 1.000 |  |
|  |  |  |  |  |  | - |  | Unrelated |  | Right |  | Controls |  | -1.684 |  | 1.457 |  | 33.7 |  | -1.1558 |  | 0.939 |  |
|  |  |  |  |  |  | - |  | Unrelated |  | Right |  | Native Signers |  | -1.852 |  | 1.839 |  | 59.1 |  | -1.0068 |  | 0.972 |  |
|  |  |  |  | Native Signers |  | - |  | Unrelated |  | Right |  | Controls |  | -2.497 |  | 1.839 |  | 59.1 |  | -1.3578 |  | 0.872 |  |
|  |  |  |  |  |  | - |  | Unrelated |  | Right |  | Native Signers |  | -2.665 |  | 1.457 |  | 33.7 |  | -1.8286 |  | 0.606 |  |
|  |  | Right |  | Controls |  | - |  | Unrelated |  | Right |  | Native Signers |  | -0.167 |  | 1.839 |  | 59.1 |  | -0.0910 |  | 1.000 |  |
|  | | | | | | | | | | | | | | | | | | | | | | | |

**Table 3: Negativity/N400 - Repeated measures ANOVA for the mean amplitude in the 200-400 ms time window**

| Within Subjects Effects | | | | | | | | | | | | | | | | | | | | | | | | | | | | | | | |
| --- | --- | --- | --- | --- | --- | --- | --- | --- | --- | --- | --- | --- | --- | --- | --- | --- | --- | --- | --- | --- | --- | --- | --- | --- | --- | --- | --- | --- | --- | --- | --- |
|  | | | | | |  | |  | | |  |  | |  | |  | | |  | | |  |  | |  | |  |  |  |  |  |
|  | | | | | | | | **Sum of Squares** | | | | **df** | | | | **Mean Square** | | | | | | ***F*** | | | ***p*** | | | **η²** | | **partial η²** | |
| Condition | | | | | |  | | 84.665 | | |  | 1 | |  | | 84.665 | | |  | | | 21.404 |  | | < .001 | |  | 0.066 |  | 0.452 |  |
| Condition ✻ Group | | | | | |  | | 10.545 | | |  | 1 | |  | | 10.545 | | |  | | | 2.666 |  | | 0.115 | |  | 0.008 |  | 0.093 |  |
| Residual | | | | | |  | | 102.842 | | |  | 26 | |  | | 3.955 | | |  | | |  |  | |  | |  |  |  |  |  |
| Hemisphere | | | | | |  | | 53.504 | | |  | 1 | |  | | 53.504 | | |  | | | 3.949 |  | | 0.058 | |  | 0.042 |  | 0.132 |  |
| Hemisphere ✻ Group | | | | | |  | | 27.646 | | |  | 1 | |  | | 27.646 | | |  | | | 2.040 |  | | 0.165 | |  | 0.022 |  | 0.073 |  |
| Residual | | | | | |  | | 352.292 | | |  | 26 | |  | | 13.550 | | |  | | |  |  | |  | |  |  |  |  |  |
| Condition ✻ Hemisphere | | | | | |  | | 8.364 | | |  | 1 | |  | | 8.364 | | |  | | | 3.464 |  | | 0.074 | |  | 0.007 |  | 0.118 |  |
| Condition ✻ Hemisphere ✻ Group | | | | | |  | | 0.792 | | |  | 1 | |  | | 0.792 | | |  | | | 0.328 |  | | 0.572 | |  | 0.001 |  | 0.012 |  |
| Residual | | | | | |  | | 62.776 | | |  | 26 | |  | | 2.414 | | |  | | |  |  | |  | |  |  |  |  |  |
| Note. Type 3 Sums of Squares | | | | | | | | | | | | | | | | | | | | | | | | | | | | | | | |
|  | | | | | | | | | | | | | | | | | | | | | | | | | | | | | | | |
| Between Subjects Effects | | | | | | | | | | | | | | | | | | | | | | | | | | | | | | | |
|  |  |  |  |  |  | |  | |  |  | | |  | |  | |  |  | |  |  | | |  | |  |  |  |  |  |  |
|  | | **Sum of Squares** | | **df** | | | **Mean Square** | | | **F** | | | | | **p** | | | **η²** | | | **partial η²** | | | | |  |  |  |  |  |  |
| Group |  | 27.8 |  | 1 |  | | 27.8 | |  | 1.31 | | |  | | 0.263 | |  | 0.022 | |  | 0.048 | | |  | |  |  |  |  |  |  |
| Residual |  | 551.9 |  | 26 |  | | 21.2 | |  |  | | |  | |  | |  |  | |  |  | | |  | |  |  |  |  |  |  |
| Note. Type 3 Sums of Squares | | | | | | | | | | | | | | | | | | | | | | | | | |  |  |  |  |  |  |

**Table 4: Negativity/N400 - Repeated measures ANOVA for the mean amplitude in the 400-600 ms time window**

| Within Subjects Effects | | | | | | | | | | | | | | | |
| --- | --- | --- | --- | --- | --- | --- | --- | --- | --- | --- | --- | --- | --- | --- | --- |
|  |  |  |  |  |  |  |  |  |  |  |  |  |  |  |  |
|  | | **Sum of Squares** | | **df** | | **Mean Square** | | ***F*** | | ***p*** | | **η²** | | **partial η²** | |
| Condition |  | 86.451 |  | 1 |  | 86.451 |  | 20.0017 |  | < .001 |  | 0.066 |  | 0.435 |  |
| Condition ✻ Group |  | 2.370 |  | 1 |  | 2.370 |  | 0.5483 |  | 0.466 |  | 0.002 |  | 0.021 |  |
| Residual |  | 112.377 |  | 26 |  | 4.322 |  |  |  |  |  |  |  |  |  |
| Hemisphere |  | 17.989 |  | 1 |  | 17.989 |  | 1.1425 |  | 0.295 |  | 0.014 |  | 0.042 |  |
| Hemisphere ✻ Group |  | 0.396 |  | 1 |  | 0.396 |  | 0.0252 |  | 0.875 |  | 0.000 |  | 0.001 |  |
| Residual |  | 409.371 |  | 26 |  | 15.745 |  |  |  |  |  |  |  |  |  |
| Condition ✻ Hemisphere |  | 4.414 |  | 1 |  | 4.414 |  | 1.0724 |  | 0.310 |  | 0.003 |  | 0.040 |  |
| Condition ✻ Hemisphere ✻ Group |  | 5.200 |  | 1 |  | 5.200 |  | 1.2635 |  | 0.271 |  | 0.004 |  | 0.046 |  |
| Residual |  | 107.013 |  | 26 |  | 4.116 |  |  |  |  |  |  |  |  |  |
| Note. Type 3 Sums of Squares | | | | | | | | | | | | | | | |
|  | | | | | | | | | | | | | | | |

| Between Subjects Effects | | | | | | | | | | | | | | | |
| --- | --- | --- | --- | --- | --- | --- | --- | --- | --- | --- | --- | --- | --- | --- | --- |
|  |  |  |  |  |  |  |  |  |  |  |  |  |  |  |  |
|  | | **Sum of Squares** | | **df** | | **Mean Square** | | ***F*** | | ***p*** | | **η²** | | **partial η²** | |
| Group |  | 0.00581 |  | 1 |  | 0.00581 |  | 2.72e-4 |  | 0.987 |  | 0.000 |  | 0.000 |  |
| Residual |  | 556.11771 |  | 26 |  | 21.38914 |  |  |  |  |  |  |  |  |  |
| Note. Type 3 Sums of Squares | | | | | | | | | | | | | | | |
