# Supplementary material for: Ongoing Sign Processing Facilitates Written Word Recognition in Deaf Native Signing Children
Source: Front Psychol. 2022 Aug 5;13:917700. doi: 10.3389/fpsyg.2022.917700 (PMC9390089; doi:10.3389/fpsyg.2022.917700)
Supplement: Supplementary file 2 [file Data_Sheet_2.docx]

**Supplementary material**

List of targets used in both experiments.

* Marks targets for which the prime fragment was ‘ambiguous’ in that it received a different completion from that of the target it was created from by at least one of four deaf native or deaf near native adult signers.

Affe (monkey)

Apfel* (apple)

Auto (car)

Ballon* (balloon)

Birne* (pear)

Blume (flower)

Boden (floor)

Brücke (bridge)

Bürste* (brush)

Butter (butter)

Drache* (dragon)

Engel* (angel)

Ente* (duck)

Fahrrad (bicycle)

Farbe (colour)

Feier (celebration)

Flasche* (bottle)

Flugzeug (airplane)

Foto (picture)

Fussball* (football/soccer)

Hammer (hammer)

Hase (rabbit)

Hexe (witch)

Honig* (honey)

Hunger (hunger)

Jacke* (coat)

Kaffee* (coffee)

Käse* (cheese)

Katze (cat)

Kirche* (church)

Kleber* (glue)

Koffer (suitcase)

Kreide* (chalk)

Kuchen (cake)

Lehrer (teacher)

Löffel (spoon)

Löwe (lion)

Mädchen (girl)

Mama* (mum)

Märchen* (fairy tale)

Messer* (knife)

Monat (month)

Motor* (engine)

Mutter (mother)

Mütze (cap)

Name (name)

Nummer (number)

Papa* (dad)

Pause (break)

Person* (person)

Pinsel* (brush)

Regen* (rain)

Roller* (scooter)

Schaufel* (shovel)

Schaukel* (swing)

Schere* (scissors)

Schlüssel (key)

Schule (school)

Schwester (sister)

Seife (soap)

Sonne (sun)

Spiegel (mirror)

Tanne (fir)

Tante* (aunt)

Tasse* (cup)

Taxi* (taxi)

Teddy* (teddy)

Teufel (devil)

Tiger* (tiger)

Treppe* (stairs)

Unfall (accident)

Urlaub (holiday)

Vater* (father)

Vogel (bird)

Wasser* (water)

Wecker (alarm)

Wiese (meadow)

Wolke (cloud)

Zeitung (newspaper)

Zirkus* (circus)
